# Cytochrome P450 monooxygenase-mediated tailoring of triterpenoids and steroids in plants

**DOI:** 10.3762/bjoc.18.135

**Published:** 2022-09-21

**Authors:** Karan Malhotra, Jakob Franke

**Affiliations:** 1 Institute of Botany, Leibniz University Hannover, Herrenhäuser Str. 2, 30419 Hannover, Germany; 2 Centre of Biomolecular Drug Research, Leibniz University Hannover, Schneiderberg 38, 30167 Hannover, Germany

**Keywords:** biosynthesis, CYPs, cytochrome P450 monooxygenases, plants, steroid, sterol, triterpene, triterpenoid

## Abstract

The cytochrome P450 monooxygenase (CYP) superfamily comprises hemethiolate enzymes that perform remarkable regio- and stereospecific oxidative chemistry. As such, CYPs are key agents for the structural and functional tailoring of triterpenoids, one of the largest classes of plant natural products with widespread applications in pharmaceuticals, food, cosmetics, and agricultural industries. In this review, we provide a full overview of 149 functionally characterised CYPs involved in the biosynthesis of triterpenoids and steroids in primary as well as in specialised metabolism. We describe the phylogenetic distribution of triterpenoid- and steroid-modifying CYPs across the plant CYPome, present a structure-based summary of their reactions, and highlight recent examples of particular interest to the field. Our review therefore provides a comprehensive up-to-date picture of CYPs involved in the biosynthesis of triterpenoids and steroids in plants as a starting point for future research.

## Introduction

Triterpenoids are a large class of natural products derived from precursors containing 30 carbon atoms and composed of six isoprene units (C_5_). The structural variety of triterpenoids found in plants is particularly astonishing, and so are their biological activities. To date, more than 20,000 different plant triterpenoids have been identified, and many of these have found applications in agronomic [1], food [2], cosmetics [3] and pharmaceutical industries [4]. Plant triterpenoids include primary metabolites such as phytosterols or steroidal hormones such as brassinosteroids, but also specialised metabolites that convey diverse biological functions [5]. A key factor for the structural richness of triterpenoids and steroids from plants lies in their extensive oxidative tailoring, which in many cases is carried out by cytochrome P450 monooxygenases (CYPs). CYPs represent one of the largest superfamilies of enzymes in plants; in many species, around 1% of all genes encode CYPs [6]. CYPs are well-known for their capacity to catalyse highly regio- and stereospecific reactions on complex substrates. Besides simple hydroxylations, they can also introduce oxo, carboxy, or epoxy moieties or double bonds. Such decorations often also enable additional layers of diversification by glycosyltransferases or acyltransferases [7]. Hence, there is considerable interest in CYPs involved in triterpenoid and steroid metabolism in plants not only for improving our understanding of plant specialised metabolism, but also for synthetic biology and chemoenzymatic synthesis. In this review, we will provide an extensive overview of CYPs involved in tailoring of triterpenoids and steroids in plants. We will first introduce the nomenclature and mechanistic properties of these enzymes, before we describe the phylogenetic distribution of triterpenoid-modifying CYPs and summarise their reaction space. Lastly, we will highlight selected recent examples of multifunctional CYPs that catalyse particularly remarkable modifications of triterpenoids. We therefore hope to provide an up-to-date overview over these key enzymes in plant triterpenoid and steroid metabolism since the last comparable endeavour from Ghosh in 2017 [7]. In addition, readers might also be interested in other excellent reviews or resources providing a more general overview over plant CYPs or CYPs from other plant pathways [6,8–13].

## Review

### Nomenclature

Considering the enormous numbers of genes encoding cytochrome P450 monooxygenases in plants, a universal naming system is crucial to group related CYPs and to facilitate functional predictions. Hence, CYPs from all kingdoms are systematically named according to their amino acid identity by the cytochrome P450 nomenclature committee (David Nelson: dnelson@uthsc.edu). CYPs are grouped into clans, families, and subfamilies. The broadest hierarchy level is represented by clans, which comprise one or multiple families. An example CYP name is CYP51G1; here, “CYP51” designates the family, “G” refers to the subfamily within the CYP51 family, and “1” represents the isoform of CYPs in this subfamily [14]. Typically, all CYPs in the same subfamily share more than 55% amino acid sequence identity, and all CYPs in the same family more than 40%, although exceptions exist [15–16]. These thresholds also underline the remarkable sequence variety of CYPs, as even enzymes with only 60–70% amino acid identity can display almost identical biochemical activity.

### Enzymatic mechanism

**Figure 1:**
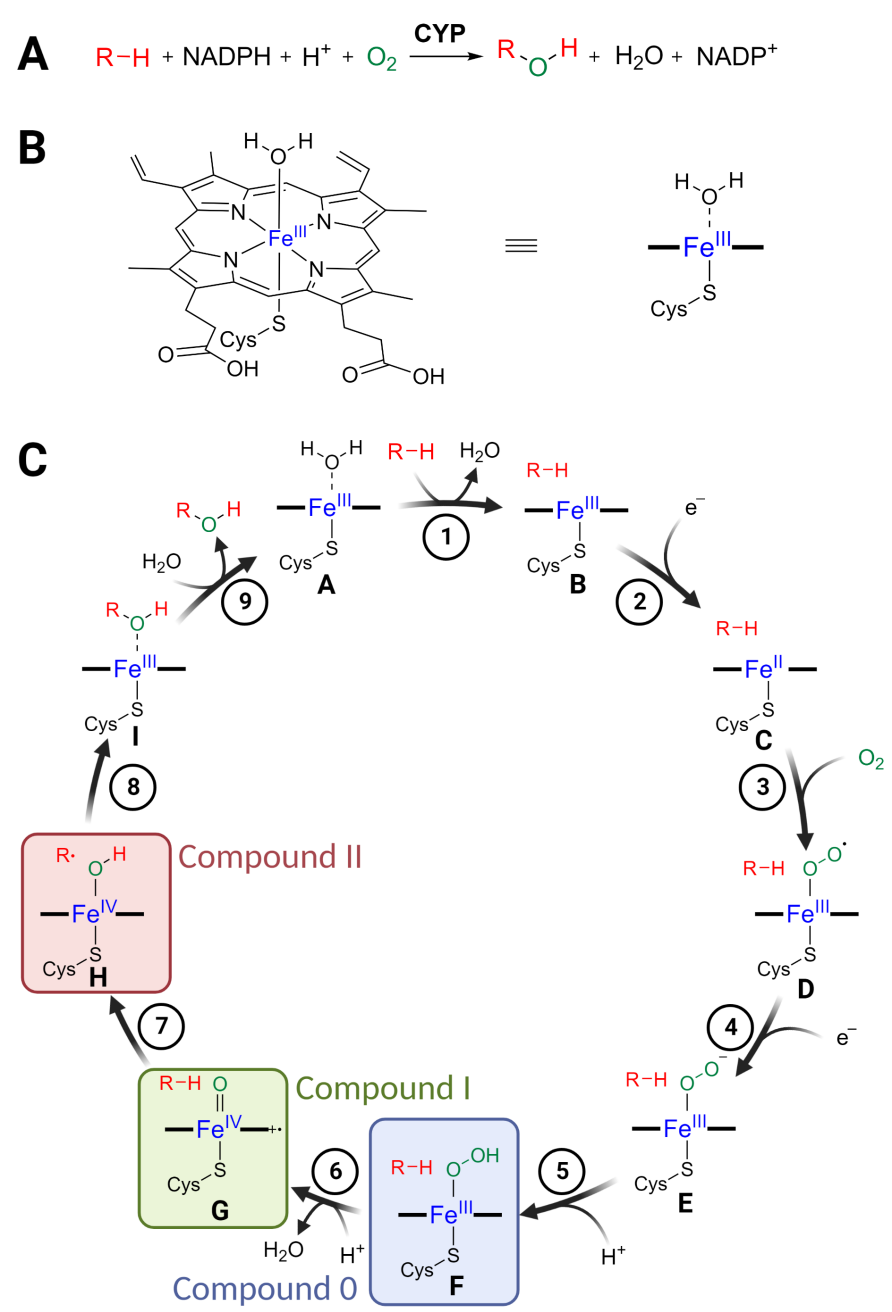
Enzyme function of cytochrome P450 monooxygenases (CYPs). A) Typical net reaction of CYPs, resulting in hydroxylation of a substrate. As monooxygenases, CYPs catalyse transfer of only one oxygen atom from molecular oxygen to their substrate. B) Heme prosthetic group containing the reactive Fe ion. Also shown is the abbreviated form of the cofactor used in the catalytic cycle. C) Catalytic cycle of CYPs. The key intermediates **F**, **G**, and **H**, called compound 0, compound I, and compound II, respectively, are highlighted. For details see main text. Figure 1 was created with BioRender.com. This content is not subject to CC BY 4.0.

As monooxygenases, CYPs catalyse the transfer of a single oxygen atom from molecular oxygen to their substrates (Figure 1A). Decades of research on CYPs led to detailed insights into their mechanistic properties based on a variety of biochemical, biophysical and computational methods [17–21]. Key for the oxidative chemistry performed by CYPs is a heme prosthetic group that activates molecular oxygen using electrons from an electron donor such as NADPH. A central Fe(III) ion is coordinated by the heme porphyrine system as well as a cysteine thiolate ligand from the protein backbone (Figure 1B). The generally accepted catalytic cycle for hydroxylations is shown in Figure 1C.

In the resting state **A**, the central ferric ion is coordinated by six ligands, four from the porphyrin ring system, one cysteine thiolate group and an aqua ligand (water), resulting in an octahedral complex [17]. The oxidative reaction is initiated by displacement of the axial water molecule by the substrate (step 1), which pushes the Fe(III) ion out of the porphyrin plane in intermediate **B** [17]. This geometrical distortion promotes electron transfer from a reductase partner protein (step 2). The most common electron donors in plants are cytochrome P450 reductases which employ NADPH for the electron transfer, but several other electron transfer systems are known [22]. The reduced ferrous intermediate **C**, bearing an overall negative charge, can then efficiently bind molecular oxygen (step 3), leading to dioxygen adduct **D**. Transfer of an additional electron from a reducing partner such as cytochrome P450 reductase (step 4) generates peroxo intermediate **E**, which upon protonation (step 5) gives hydroperoxo intermediate **F**, also called compound 0. This nucleophilic and basic intermediate is prone to dehydration (step 6), leading to the strongly electrophilic and oxidising key intermediate **G**, which is commonly known as compound I (cpd I). Although there has been a lot of debate regarding the exact structure and electronic properties of compound I (intermediate **G**), it is now generally accepted as a ferryl (Fe(IV)) oxo species with a radical cation in the porphyrin system [18,23]. In the case of hydroxylations, the oxygen from compound I (intermediate **G**) can then be transferred by an oxygen rebound mechanism (steps 7 and 8) via the ferryl hydroxy intermediate **H**, also known as compound II. This leads to the hydroxylated product coordinated to ferric ion (intermediate **I**). Lastly, displacement of the product by water regenerates the resting state **A** (step 9). In addition to simple hydroxylations, slight deviations from this general mechanistic cycle can also lead to different reaction outcomes, e.g., rearrangements, desaturations or epoxidations. Multiple oxidation rounds, leading to aldehydes/ketones or carboxylic acids, are also commonly observed. Together, this versatile oxidative chemistry makes CYPs key enzymes in specialised metabolism in general [8,11], and crucial agents for the structural diversity of triterpenes.

### Phylogenetic distribution of triterpenoid- and steroid-modifying plant cytochrome P450 monooxygenases

**Figure 2:**
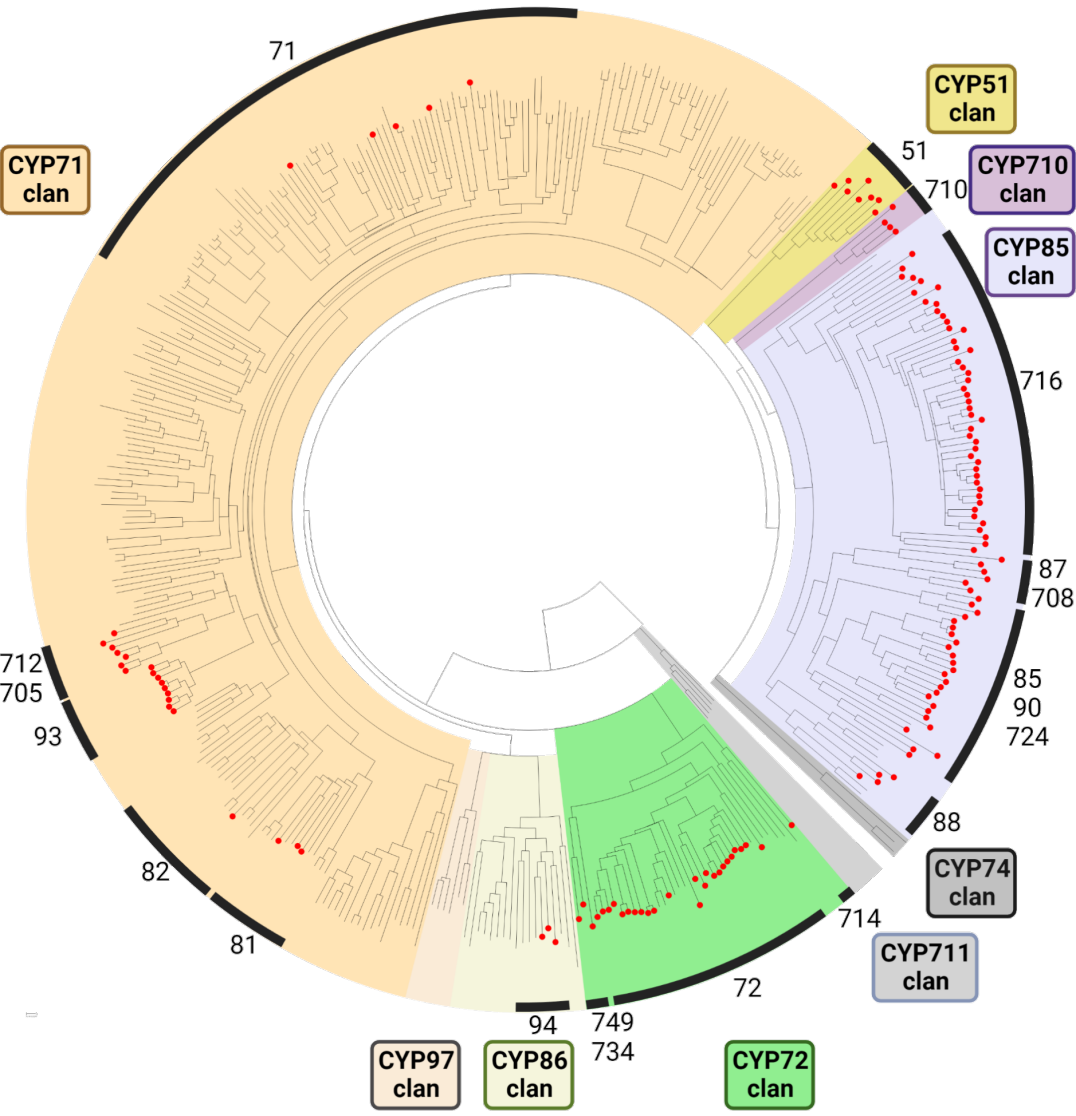
Phylogenetic distribution of CYPs acting on triterpenoid and steroid scaffolds (red nodes) compared to other CYPs from higher plants [15]. Numbers and black bars mark CYP families containing CYPs known to act on triterpenoids and steroids. Amino acid sequences (149 triterpenoid CYPs, 266 non-triterpenoid CYPs) were aligned using MUSCLE [92], and a neighbour-joining consensus tree of 1,000 bootstrap replicates was generated using the Jukes–Cantor model. The final tree was visualised in Python using the ete3 package. Annotations in Figure 2 were created with BioRender.com. This content is not subject to CC BY 4.0. A high resolution version with tip labels is available as Supporting Information File 1.

To assess the phylogenetic distribution of triterpenoid-modifying CYPs in comparison to other CYPs from plants, we collected a total of 149 CYPs from plants reported in the literature which are involved in triterpenoid or steroid metabolism (Table 1), and generated a neighbour-joining tree together with 266 non-triterpenoid CYPs with a different substrate scope (Figure 2). Notably, our analysis highlights that triterpenoid CYPs do not seem to occur randomly in various CYP clans and families; instead, certain clans and families represent “hotspots” of triterpenoid/steroid-modifying CYPs.

**Table 1:** List of characterised plant cytochrome P450 monooxygenases (CYPs) modifying triterpenoids or steroids.

Name	Clan	Family	Species	Accession number	Scaffold	Substrate	Reaction	Product	Ref.

CYP51G1	51	51	*Sorghum bicolor*	XM_0214610212.1	steroid	obtusifoliol	C14α demethylation	4α-methyl- 5α-ergosta-8, 14,24(28)- trien-3β-ol	[24]
CYP51G1	51	51	*Arabidopsis thaliana*	AB014459	steroid	obtusifoliol	C14α demethylation	4α-methyl- 5α-ergosta-8, 14,24(28)- trien-3β-ol	[25]
CYP51H10	51	51	*Avena strigosa*	DQ680852	pentacyclic oleanane	β-amyrin	C12–C13β epoxidation / C16 β hydroxlation	12,13-β- epoxy-16-β- hydroxy- amyrin	[1]
CYP51H14	51	51	*Brachy-* *podium distachyon*	ON108677	pentacyclic triterpene	19-hydroxy- isoarborinol	C7 and C28 hydroxylation	7,19,28-tri- hydroxyiso- arborinol	[26]
CYP51H15	51	51	*Brachy-* *podium distachyon*	ON108678	pentacyclic triterpene	isoarborinol	C19 hydroxylation	19-hydroxy- isoarborinol	[26]
CYP51H16	51	51	*Brachy-* *podium distachyon*	ON108679	pentacyclic triterpene	7,19,28-tri- hydroxyiso- arborinol	C1 hydroxylation	1,7,19,28- tetrahydroxy- isoarborinol	[26]
CYP51H35	51	51	*Triticum aestivum*	ON108669	pentacyclic triterpene	isoarborinol	C19 hydroxylation	19-hydroxy- isoarborinol	[26]
CYP51H37	51	51	*Triticum aestivum*	ON108670	pentacyclic triterpene	19-hydroxy- isoarborinol	C25 hydroxylation and C2 oxidation	ellarinacin	[26]
CYP71A16	71	71	*Arabidopsis thaliana*	NM_123623.5	monocyclic triterpene aldehyde	marneral / marnerol	C23 hydroxylation	23-hydroxy- marneral / 23-hydroxy- marnerol	[27–28]
CYP71BQ5	71	71	*Melia azedarach*	MK803264.1	tirucallane triterpenoid	dihydroniloticin	C21 hydroxylation	melianol	[29]
CYP71CD2	71	71	*Melia azedarach*	MK803271	tirucallane triterpenoid	tirucalla-7,24- dien-3β-ol	C23 hydroxylation and C24–C25 epoxidation	dihydro- niloticin	[29]
CYP71D353	71	71	*Lotus japonicus*	KF460438	pentacyclic lupane	dihydrolupeol / 20-hydroxy- lupeol	C20 hydroxylation / C28 oxidation	20-hydroxy- lupeol / 20-hydroxy- betulinic acid	[30]
CYP71D443	71	71	*Ajuga reptans*	LC066937	steroid	3β-hydroxy-5β- cholestan-6-one	C22 hydroxylation	3β,22*R*- dihydroxy-5β- cholestan-6- one	[31]
CYP81AQ19	71	81	*Momordica charantia*	LC456843	tetracyclic triterpenoid	cucurbitadienol	C23α hydroxylation	cucurbita- 5,24-dien- 3,23α-diol	[32]
CYP81Q58	71	81	*Cucumis sativus*	KM655856	tetracyclic triterpenoid	19-hydroxy- cucurbitadienol	C25 hydroxylation / double bond shift	19,25- dihydroxy- cucurbita- dienol	[33]
CYP81Q59	71	81	*Cucumis melo*	Melo3C022375	tetracyclic triterpenoid	11-carbonyl- 20β-hydroxy- cucurbitadienol	C2β hydroxylation	11-carbonyl- 2β,20β- dihydroxy- cucurbita dienol	[34]
CYP82J17	71	82	*Trigonella foenum-* *graecum*	MK636709	steroid	16*S*-hydroxy- 22-oxo- cholesterol	C27 hydroxy- lation / spiro- ketalisation	diosgenin	[35]
CYP93A220 / IaAO5	71	93	*Ilex asprella*	MZ508433	pentacyclic oleanane	β-amyrin	C24 oxidation	α-boswellic acid	[36]
CYP93E1	71	93	*Glycine max*	AB231332	pentacyclic oleanane	β-amyrin / sophoradiol	C24 hydroxylation	24-hydroxy- β-amyrin / soyasapo- genol B	[37]
CYP93E2	71	93	*Medicago truncatula*	DQ335790	pentacyclic oleanane	β-amyrin	C24 hydroxylation	24-hydroxy- β-amyrin	[38]
CYP93E3	71	93	*Glycyrrhiza uralensis*	AB437320	pentacyclic oleanane	β-amyrin	C24 hydroxylation	24-hydroxy- β-amyrin	[39]
CYP93E4	71	93	*Arachis hypogaea*	KF906535	pentacyclic oleanane	β-amyrin	C24 hydroxylation	24-hydroxy- β-amyrin	[40]
CYP93E5	71	93	*Cicer arietinum*	KF906536	pentacyclic oleanane	β-amyrin	C24 hydroxylation	24-hydroxy- β-amyrin	[40]
CYP93E6	71	93	*Glycyrrhiza glabra*	KF906537	pentacyclic oleanane	β-amyrin	C24 hydroxylation	24-hydroxy- β-amyrin	[40]
CYP93E7	71	93	*Lens culinaris*	KF906538	pentacyclic oleanane	β-amyrin	C24 hydroxylation	24-hydroxy- β-amyrin	[40]
CYP93E8	71	93	*Pisum sativum*	KF906539	pentacyclic oleanane	β-amyrin	C24 hydroxylation	24-hydroxy- β-amyrin	[40]
CYP93E9	71	93	*Phaseolus vulgaris*	KF906540	pentacyclic oleanane	β-amyrin	C24 hydroxylation	24-hydroxy- β-amyrin	[40]
CYP705A1	71	705	*Arabidopsis thaliana*	NM_001341032.1	tricyclic triterpenoid	arabidiol	C15–C16 cleavage	14-apo- arabidiol	[28]
CYP705A5	71	705	*Arabidopsis thaliana*	NM_124173.3	tricyclic triterpenoid	7β-hydroxy- thalianol	C15–C16 desaturation	desaturated (C15–C16) 7β-hydroxy- thalianol	[41]
CYP712K1	71	712	*Tripterygium wilfordii*	MN621243	pentacyclic triterpenoid	friedelin	C29 oxidation	polpunonic acid and 29-hydroxy- friedelin	[42]
CYP712K2	71	712	*Tripterygium wilfordii*	MN621244	pentacyclic triterpenoid	friedelin	C29 oxidation	polpunonic acid and 29-hydroxy- friedelin	[42]
CYP712K3	71	712	*Tripterygium wilfordii*	MN621245	pentacyclic triterpenoid	friedelin	C29 oxidation	polpunonic acid and 29-hydroxy- friedelin	[42]
CYP712K4	71	712	*Maytenus ilicifolia*	MK829814	pentacyclic triterpenoid	friedelin	C29 oxidation	polpunonic acid or maytenoic acid	[43]
CYP72A61	72	72	*Medicago truncatula*	DQ335793	pentacyclic oleanane	24-hydroxy- β-amyrin	C22 hydroxylation	soyasapo- genol B	[44]
CYP72A61v2	72	72	*Medicago truncatula*	XM_003605422	pentacyclic oleanane	24-hydroxy- β-amyrin	C22 hydroxylation	soyasapo- genol B	[44]
CYP72A62v2	72	72	*Medicago truncatula*	AB558147	pentacyclic oleanane	β-amyrin	C29 oxidation	29-hydroxy- β-amyrin / epi-katonic acid	[45]
CYP72A63	72	72	*Medicago truncatula*	AB558146	pentacyclic oleanane	β-amyrin	C30 oxidation	11-deoxy- glycyrrhen- tinic acid	[46]
CYP72A64v2	72	72	*Medicago truncatula*	MK534548	pentacyclic oleanane	β-amyrin	C29 oxidation	29-hydroxy- β-amyrin / epi-katonic acid	[45]
CYP72A65v2	72	72	*Medicago truncatula*	XM_003628012.4	pentacyclic oleanane	β-amyrin	C21 hydroxylation	21-hydroxy- β-amyrin	[45]
CYP72A67	72	72	*Medicago truncatula*	DQ335780	pentacyclic oleanane	oleanolic acid / hederagenin / gypsogenic acid / gypsogenin	C2β hydroxylation	2β-hydroxy- oleanolic acid / bayogenin / medicagenic acid / 2β,3β- dihydroxy- olean-12-en- 23-oxo-28- oic acid	[47–48]
CYP72A68	72	72	*Medicago truncatula*	DQ335782	pentacyclic oleanane	oleanolic acid / hederagenin / gypsogenin	C23 oxidation	hederagenin / gypsogenin / gypsogenic acid	[48]
CYP72A68v2	72	72	*Medicago truncatula*	XM_013608494.3	pentacyclic oleanane	oleanolic acid / hederagenin / gypsogenin	C23 oxidation	hedera- genin / gypsogenin / gypsogenic acid	[44]
CYP72A69	72	72	*Glycine max*	LC143440	pentacyclic oleanane	soyasapogenol B	C21 hydroxylation	soyasapo- genol A	[49]
CYP72A141	72	72	*Glycine max*	MK534532	pentacyclic oleanane	β-amyrin	C29 hydroxylation	29-hydroxy- β-amyrin	[45]
CYP72A154	72	72	*Glycyrrhiza uralensis*	AB558153	pentacyclic oleanane	β-amyrin / 11-oxo-β-amyrin	C30 oxidation	30-hydroxy- β-amyrin / glycyrrhetinic acid	[46]
CYP72A302	72	72	*Phaseolus vulgaris*	MK534537	pentacyclic oleanane	β-amyrin	C29 hydroxylation	29-hydroxy- β-amyrin	[45]
CYP72A397	72	72	*Kalopanax septemlobus*	KT150517	pentacyclic oleanane	oleanolic acid	C23 oxidation	hederagenin	[50]
CYP72A552	72	72	*Barbarea vulgaris*	MH252571	pentacyclic oleanane	oleanolic acid	C23 oxidation	hederagenin	[51]
CYP72A557	72	72	*Medicago truncatula*	MK534544	pentacyclic oleanane	β-amyrin	C21 hydroxylation	21-hydroxy- β-amyrin	[45]
CYP72A558	72	72	*Medicago truncatula*	MK534545	pentacyclic oleanane	β-amyrin	C21 hydroxylation	21-hydroxy- β-amyrin	[45]
CYP72A559	72	72	*Medicago truncatula*	MK534546	pentacyclic oleanane	β-amyrin	C21 hydroxylation	21-hydroxy- β-amyrin	[45]
CYP72A613	72	72	*Trigonella foenum-* *graecum*	MK636708	steroid	16*S*-hydroxy- 22-oxo- cholesterol	C27 hydroxylation / spiro- ketalisation	diosgenin	[35]
CYP72A616	72	72	*Paris polyphylla*	MK636705	steroid	16*S*-hydroxy- 22-oxo- cholesterol	C27 hydroxy- lation / spiro- ketalisation	diosgenin	[35]
CYP72A694	72	72	*Vigna angularis*	MK534538	pentacyclic oleanane	β-amyrin / 29-hydroxy- β-amyrin	C29 oxidation	29-hydroxy- β-amyrin / epi-katonic acid	[45]
CYP72A697	72	72	*Lotus japonicus*	MK534539	pentacyclic oleanane	β-amyrin	C29 hydroxylation	29-hydroxy- β-amyrin	[45]
CYP72A699	72	72	*Trifolium pratense*	MK534549	pentacyclic oleanane	β-amyrin / 29-hydroxy- β-amyrin	C29 oxidation	29-hydroxy- ß-amyrin / epi-katonic acid	[45]
CYP714E19	72	714	*Centella asiatica*	KT004520	pentacyclic oleanane / ursane	oleanolic acid / ursolic acid	C23 oxidation	hederagenin / 23- hydroxy- ursolic acid	[52]
CYP714E88 / IaAO4	72	714	*Ilex asprella*	MZ508437	pentacyclic oleanane / ursane	ursolic acid / oleanolic acid	C23 oxidation	23-carboxyl- ursolic acid / gypsogenic acid	[36]
CYP734A7	72	734	*Solanum lycoper-* *sicum*	AB223041	steroid	castasterone / 28-nor- castasterone / brassinolide	C26 hydroxylation	26-hydroxy- castaster- one / 26-hydroxy- norcastaster- one / 26-hydroxy- brassinolide	[53]
CYP749A63	72	749	*Crataegus pinnatifida*	MF596155	pentacyclic oleanane	oleanolic acid	C24 hydroxylation	4-epi- hederagenin	[54]
CYP85A1	85	85	*Arabidopsis thaliana*	AB035868	steroid	6-deoxoteaster- one / 3-dehydro- 6-deoxoteaster- one / 6-deoxo- typhasterol / 6-deoxo- castasterone	C6 oxidation	teasterone / 3-dehydro- teasterone / typhasterol / castasterone	[55]
CYP85A1	85	85	*Solanum lycoper-* *sicum*	U54770	steroid	6-deoxoteaster- one / 3-dehydro- 6-deoxoteaster- one / 6-deoxo- typhasterol / 6-deoxo- castasterone	C6 oxidation	teasterone / 3-dehydro- teasterone / typhasterol / castasterone	[56]
CYP85A2	85	85	*Arabidopsis thaliana*	AB087801	steroid	castasterone / 6-deoxo- castaster- one / 6-deoxo- typhasterol / 3-dehydro-6- deoxo- teaster- one	Baeyer- Villiger oxidation / C6 oxidation	brassinolide / castaster- one / typhasterol / 3-dehydro- teasterone	[57–58]
CYP85A3	85	85	*Solanum lycoper-* *sicum*	AB190445	steroid	6-deoxocastas- terone / castasterone	Baeyer- Villiger oxidation / C6 oxidation	castasterone / brassinolide	[58]
CYP87D16	85	87	*Maesa lanceolata*	KF318735	pentacyclic oleanane	β-amyrin	C16α hydroxylation	16α-hydroxy- β-amyrin	[59]
CYP87D18	85	87	*Siraitia grosvenorii*	HQ128570	tetracyclic triterpenoid	cucurbitadienol / 11α-hydroxy- cucurbitadienol/ 24,25-di- hydroxy- cucurbitadienol	C11 oxidation	11α-hydroxy- cucurbita- dienol / 11-oxo- cucurbita- dienol / mogrol	[34]
CYP87D20	85	87	*Cucumis sativus*	Csa1G044890	tetracyclic triterpenoid	cucurbitadienol / 11-oxo-cucur- bitadienol	C11 oxidation / C20β hydroxylation	11-oxocucur- bitadienol / 11-carbonyl- 20β-hydroxy- cucurbita- dienol	[34]
CYP88D6	85	88	*Glycyrrhiza uralensis*	AB433179	pentacyclic oleanane	β-amyrin	C11 oxidation	11-oxo-β- amyrin	[39]
CYP88L2	85	88	*Cucumis sativus*	Csa3G903540	tetracyclic triterpenoid	cucurbitadienol / 11-oxo-cucur- bitadienol	C19 hydroxylation	19-hydroxy- cucurbita- dienol	[34]
CYP88L7	85	88	*Momordica charantia*	LC456844	tetracyclic triterpenoid	cucurbitadienol	C19 hydroxylation, C5 and C19 ether bridge	cucurbita- 5,24-dien- 3β,19-diol and 5β,19- epoxy- cucurbita- 6,24-dien- 3β-ol	[32]
CYP88L8	85	88	*Momordica charantia*	LC456845	tetracyclic triterpenoid	cucurbitadienol	C7β hydroxylation	cucurbita- 5,24-dien- 3β,7β-diol	[32]
CYP90A1	85	90	*Arabidopsis thaliana*	X87367	steroid	6-deoxocat- hasterone / 6-deoxoteaster- one / 22*S*-hydroxy- campesterol / 22*R*,23*R*-di- hydroxycam- pesterol	C3 oxidation	22*S*-hydroxy- 5α-campes- tan-3-one / 3-dehydro-6- deoxo- teasterone/ 22*S*-hydroxy- campest-4- en-3-one / 22*R*,23*R*- dihydroxy- campest-4- en-3-one	[60]
CYP90B1	85	90	*Arabidopsis thaliana*	NM_114926.4	steroid	campesterol / 24*R*-ergost-4- en-3-one / 24*R*-5α-ergos- tan-3-one / campestanol / 6-oxocampes- tanol	C22 hydroxylation	22*S*-hydroxy- campesterol / 22*S*-hydroxy- 24*R*-ergost- 4-en-3-one / 22*S*-hydroxy- 24*R-* 5α- ergostan-3- one / 6- deoxocat- hasterone / cathasterone	[61]
CYP90B2	85	90	*Oryza sativa*	AB206579	steroid	campesterol / campestanol	C22 hydroxylation	22*S*-hydroxy- campesterol / 6-deoxo- cathasterone	[62]
CYP90B3	85	90	*Solanum lycoper-* *sicum*	NM_001279330.2	steroid	campesterol / 24*R*-ergost-4- en-3-one / 24*R*-5α-ergos- tan-3-one / campestanol	C22 hydroxylation	22-hydroxy- campesterol / 22*S*-hydroxy- 24*R-*ergost- 4-en-3-one / 22*S*-hydroxy- 24R-5α- ergostan-3- one / 6-deoxo- cathasterone	[63]
CYP90B27	85	90	*Veratrum californicum*	KJ869252	steroid	cholesterol / 26-hydroxy- cholesterol / 7ß-hydroxy- cholesterol	C22 hydroxylation	22*R*-hydroxy- cholesterol / 22,26-di- hydroxy- cholesterol / 7ß,22-di- hydroxy- cholesterol	[64]
CYP90B27	85	90	*Paris polyphylla*	KX904822	steroid	cholesterol	C22 hydroxylation	22*R*-hydroxy- cholesterol	[65]
CYP90B50	85	90	*Trigonella foenum-* *graecum*	MK636707	steroid	cholesterol	C22*R*, C16 dihydroxy- lation	16*S*,22*R*- dihydroxy- cholesterol	[35]
CYP90B51	85	90	*Trigonella foenum-* *graecum*	MK636706	steroid	cholesterol	C22*S* hydroxylation / C22*R* hydroxylation	22*S*-hydroxy- cholesterol / 22*R*-hydroxy- cholesterol	[66]
CYP90B52	85	90	*Paris polyphylla*	MK636701	steroid	cholesterol	C22*S* hydroxylation	22*S*-hydroxy- cholesterol	[35]
CYP90B71	85	90	*Dioscorea* *zingi-* *berensis*	MN829441	steroid	cholesterol	C22*R* hydroxylation	22*R*-hydroxy- cholesterol	[66]
CYP90C1	85	90	*Arabidopsis thaliana*	NM_001342408.1	steroid	22*S*-hydroxy- 24*R*-5α-ergost- an-3-one / 3-epi-6- deoxocat- hasterone	C23 hydroxylation	3-dehydro-6- deoxoteas- terone / 6-deoxo- typhasterol	[67]
CYP90D1	85	90	*Arabidopsis thaliana*	NM_112223	steroid	22*S*-hydroxy- 24*R*-5α-ergost- an-3-one / 3-epi-6- deoxocat- hasterone	C23 hydroxylation	3-dehydro-6-deoxoteas- terone / 6-deoxo- typhasterol	[67]
CYP90D2	85	90	*Oryza sativa*	NM_001409071	steroid	22*S*-hydroxy- 24*R*-5α-ergost- an-3-one / 3-epi-6- deoxocat- hasterone	C23 hydroxylation	3-dehydro-6- deoxoteas- terone / 6-deoxo- typhasterol	[68]
CYP90D3	85	90	*Oryza sativa*	AAT44310	steroid	22*S*-hydroxy- 24*R*-5α-ergost- an-3-one / 3-epi-6- deoxocat- hasterone	C23 hydroxylation	3-dehydro-6- deoxoteas- terone / 6-deoxo- typhasterol	[68]
CYP90G1v1	85	90	*Veratrum californicum*	KJ869258	steroid	22*R*-hydroxy- cholesterol / 22,26-di- hydroxycholes- terol / 22-hydroxy-26- aminocholes- terol	C22 hydroxylation	22-keto- cholesterol / 22-keto-26- hydroxy- cholesterol / verazine	[64]
CYP90G1v2	85	90	*Veratrum californicum*	KJ869261	steroid	22*R*-hydroxy- cholesterol / 22,26-di- hydroxycholes- terol / 22-hydroxy-26- aminocholes- terol	C22 hydroxylation	22-keto- cholesterol / 22-keto-26- hydroxy- cholesterol / verazine	[64]
CYP90G1v3	85	90	*Veratrum californicum*	KJ869260	steroid	22*R*-hydroxy- cholesterol / 22,26-di- hydroxycholes- terol / 22-hydroxy-26- aminocholes- terol	C22 hydroxylation	22-keto- cholesterol / 22-keto-26- hydroxy- cholesterol / verazine	[64]
CYP90G4	85	90	*Paris polyphylla*	MK636702	steroid	22*R*-hydroxy- cholesterol	C16 oxidation	16*S*,22*R*- dihydroxy- cholesterol	[66]
CYP90G6	85	90	*Dioscorea* *zingi-* *berensis*	MN829442	steroid	22*R*-hydroxy- cholesterol	C16 oxidation	16*S*,22*R*- dihydroxy- cholesterol	[66]
CYP708A15	85	708	*Iberis amara*	MW514550	tetracyclic triterpenoid	16β-hydroxy- cucurbitadienol	C22 hydroxylation	16β,22- dihydroxy- cucurbita- dienol	[69]
CYP708A15v2	85	708	*Iberis amara*	MW514551	tetracyclic triterpenoid	16β-hydroxy- cucurbitadienol	C22 hydroxylation	16β,22- dihydroxy- cucurbita- dienol	[69]
CYP708A16	85	708	*Iberis amara*	MW514556	tetracyclic triterpenoid	cucurbitadienol	C16 hydroxylation	16β-hydroxy- cucurbita- dienol	[69]
CYP708A2	85	708	*Arabidopsis thaliana*	NM_001344756.1	tricyclic triterpenoid	thalianol	C7β hydroxylation	7β-hydroxy- thalianol	[41]
CYP716A1	85	716	*Arabidopsis thaliana*	NM_123002.2	pentacyclic ursane / oleanane / lupane	α-amyrin / β-amyrin / lupeol	C28 oxidation	ursolic acid / oleanolic acid / betulin	[70]
CYP716A2	85	716	*Arabidopsis thaliana*	LC106013.1	pentacyclic ursane / oleanane / lupane	α-amyrin / β-amyrin / lupeol	C16/C22α/ C28 hydroxylation	uvaol / C22α- hydroxy-β- amyrin / erythrodiol / betulin	[70]
CYP716A12	85	716	*Medicago truncatula*	FN995113	pentacyclic ursane / oleanane / lupane	α-amyrin / β-amyrin / betulin	C28 oxidation	ursolic acid / oleanolic acid / betulinic acid	[71]
CYP716A14v2	85	716	*Artemisia annua*	KF309251	pentacyclic ursane / oleanane	α-amyrin / β-amyrin	C3 oxidation	α-amyrone / β-amyrone	[72]
CYP716A15	85	716	*Vitis vinifera*	AB619802	pentacyclic ursane / oleanane / lupane	α-amyrin / β-amyrin / betulin	C28 oxidation	ursolic acid / oleanolic acid / betulinic acid	[73]
CYP716A17	85	716	*Vitis vinifera*	AB619803	pentacyclic oleanane	β-amyrin	C28 oxidation	oleanolic acid	[73]
CYP716A44	85	716	*Solanum* *lycoper-* *sicum*	XM_004239248.4	pentacyclic ursane / oleanane	α-amyrin / β-amyrin	C28 oxidation	ursolic acid / oleanolic acid	[74]
CYP716A46	85	716	*Solanum* *lycoper-* *sicum*	XM_004243858	pentacyclic ursane / oleanane	α-amyrin / β-amyrin	C28 oxidation	ursolic acid / oleanolic acid	[74]
CYP716A51	85	716	*Lotus japonicus*	AB706297	pentacyclic ursane / oleanane / lupane	α-amyrin / β-amyrin / lupeol	C28 oxidation	ursolic acid / oleanolic acid / betulinic acid	[75]
CYP716A52v2	85	716	*Panax ginseng*	JX036032	pentacyclic oleanane	β-amyrin	C28 oxidation	oleanolic acid	[76]
CYP716A75	85	716	*Maesa lanceolata*	KF318733	pentacyclic oleanane	β-amyrin	C28 oxidation	oleanolic acid	[59]
CYP716A78	85	716	*Cheno-* *podium* *quinoa*	KX343075	pentacyclic oleanane	β-amyrin	C28 oxidation	oleanolic acid	[77]
CYP716A79	85	716	*Cheno-* *podium* *quinoa*	KX343076	pentacyclic oleanane	β-amyrin	C28 oxidation	oleanolic acid	[77]
CYP716A80	85	716	*Barbarea vulgaris*	KP795926	pentacyclic ursane / oleanane / lupane	α-amyrin / β-amyrin / lupeol	C28 oxidation	ursolic acid / oleanolic acid / betulinic acid	[78]
CYP716A81	85	716	*Barbarea vulgaris*	KP795925	pentacyclic ursane / oleanane / lupane	α-amyrin / β-amyrin / lupeol	C28 oxidation	ursolic acid / oleanolic acid / betulinic acid	[78]
CYP716A83	85	716	*Centella asiatica*	KU878849	pentacyclic ursane / oleanane	α-amyrin / β-amyrin	C28 oxidation	ursolic acid / oleanolic acid	[79]
CYP716A86	85	716	*Centella asiatica*	KU878848	pentacyclic oleanane	β-amyrin	C28 oxidation	oleanolic acid	[79]
CYP716A94	85	716	*Kalopanax septemlobus*	KT150521	pentacyclic oleanane	β-amyrin	C28 oxidation	oleanolic acid	[50]
CYP716A110	85	716	*Aquilegia coerulea*	KU878864	pentacyclic oleanane	β-amyrin	C28 oxidation	oleanolic acid	[79]
CYP716A111	85	716	*Aquilegia coerulea*	KY047600	pentacyclic oleanane	β-amyrin	C16β hydroxylation	16β-hydroxy- β-amyrin	[79]
CYP716A113	85	716	*Aquilegia coerulea*	KU878866	tetracyclic triterpenoid	cycloartenol	unknown regio- selectivity	hydroxy- cyclo- artenol, performs non- specific reaction of endogenous yeast compounds	[79]
CYP716A140	85	716	*Platycodon grandiflorus*	KU878853	pentacyclic oleanane / ursane	β-amyrin / 16β-hydroxy- β-amyrin / 12,13α-epoxy- β-amyrin	C28 oxidation	oleanolic acid / 16β-hydroxy- oleanolic acid	[79]
CYP716A140v2	85	716	*Platycodon grandiflorus*	LC209199	pentacyclic oleanane	β-amyrin	C28 oxidation	oleanolic acid	[80]
CYP716A141	85	716	*Platycodon grandiflorus*	KU878855	pentacyclic oleanane	β-amyrin / oleanolic acid	C28 oxidation / C16β hydroxylation	oleanolic acid / 16β-hydroxy- oleanolic acid	[79–80]
CYP716A154	85	716	*Catharan-* *thus roseus*	JN565975	pentacyclic ursane / oleanane / lupane	α-amyrin / β-amyrin / betulin	C28 oxidation	ursolic acid / oleanolic acid / betulinic acid	[81]
CYP716A155	85	716	*Rosmarinus officinalis*	MK592859	pentacyclic lupane	lupeol	C28 oxidation	betulinic acid	[82]
CYP716A175	85	716	*Malus domestica*	XM_008392874	pentacyclic ursane / oleanane / lupane	α-amyrin / β-amyrin / lupeol / germanicol	C28 oxidation	ursolic acid / oleanolic acid / betulinic acid / morolic acid	[83]
CYP716A179	85	716	*Glycyrrhiza uralensis*	LC157867	pentacyclic ursane / oleanane / lupane	α-amyrin / β-amyrin / betulin	C28 oxidation / C22α hydroxylation	ursolic acid / C22α- hydroxy- amyrin / oleanolic aicd / betulinic acid	[84]
CYP716A180	85	716	*Betula platyphylla*	KJ452328	pentacyclic lupane	lupeol	C28 oxidation	betulinic acid	[85]
CYP716A210 / IaAO1	85	716	*Ilex asprella*	MK994507	pentacyclic ursane / oleanane	α-amyrin / β-amyrin	C28 oxidation	ursolic acid / oleanolic acid	[86]
CYP716A244	85	716	*Eleuthero-* *coccus* *senticosus*	KX354739	pentacyclic oleanane	β-amyrin	C28 oxidation	oleanolic acid	[87]
CYP716A252	85	716	*Ocimum basilicum*	JQ958967	pentacyclic ursane / oleanane	α-amyrin / β-amyrin	C28 oxidation	ursolic acid / oleanolic acid	[88]
CYP716A253	85	716	*Ocimum basilicum*	JQ958968	pentacyclic ursane / oleanane	α-amyrin / β-amyrin	C28 oxidation	ursolic acid / oleanolic acid	[88]
CYP716A265	85	716	*Lager-* *stroemia* *speciosa*	MG708187	pentacyclic ursane / oleanane / lupane	α-amyrin / β-amyrin / lupeol	C28 oxidation	ursolic acid / oleanolic acid / betulinic acid	[89]
CYP716A266	85	716	*Lager-* *stroemia* *speciosa*	MG708188	pentacyclic ursane / oleanane / lupane	α-amyrin / β-amyrin / lupeol	C28 oxidation	ursolic acid / oleanolic acid / betulinic acid	[89]
CYP716C11	85	716	*Centella asiatica*	KU878852	pentacyclic oleanane / ursane	oleanolic acid / ursolic acid / 6β-hydroxy- oleanolic acid	C2α hydroxylation	maslinic acid / 2α-hydroxy- ursolic acid / 6β-hydroxy- maslinic acid	[79]
CYP716C49	85	716	*Crataegus pinnatifida*	MF120282	pentacyclic oleanane / ursane / lupane	oleanolic acid / ursolic acid / betulinic acid	C2α hydroxylation	maslinic acid / corosolic acid / alphitolic acid	[54]
CYP716C55	85	716	*Lager-* *stroemia speciosa*	MG708191	pentacyclic ursane / oleanane	ursolic acid / oleanolic acid	C2α hydroxylation	corosolic acid / maslinic acid	[89]
CYP716E26	85	716	*Solanum lycoper-* *sicum*	XM_004241773	pentacyclic ursane / oleanane	α-amyrin / β-amyrin	C6β hydroxylation	6β-hydroxy- α-amyrin / daturadiol	[74]
CYP716E41	85	716	*Centella asiatica*	KU878851	pentacyclic oleanane / ursane	oleanolic acid / ursolic acid / maslinic acid	C6β hydroxylation	6β-hydroxy- oleanolic acid / 6β-hydroxy- ursolic acid / 6β-hydroxy- maslinic acid	[79]
CYP716S1	85	716	*Panax ginseng*	JX036031	tetracyclic triterpene	protopanaxadiol	C6 hydroxylation	protopanaxa- triol	[76]
CYP716S5	85	716	*Platycodon grandiflorus*	KU878856	pentacyclic oleanane	β-amyrin / oleanolic acid	C12-C13α epoxidation	C12-C13α- epoxy-β- amyrin / C12-C13α- epoxy- oleanolic acid	[79]
CYP716U1	85	716	*Panax ginseng*	JN604536	tetracyclic triterpene	dammarene- diol-II	C12 hydroxylation	protopanaxa- diol	[76]
CYP716Y1	85	716	*Bupleurum falcatum*	KC963423	pentacyclic ursane / oleanane	α-amyrin / β-amyrin	C16α hydroxylation	16α-hydroxy- α-amyrin / 16α-hydroxy- β-amyrin	[38]
IaAO2	85	716	*Ilex asprella*	OL604227	pentacyclic ursane / oleanane	α-amyrin / β-amyrin	C28 oxidation	ursolic acid / oleanolic acid	[36]
CYP724A1	85	724	*Arabidopsis thaliana*	NM_001343334.1	steroid	possibly brassinosteroids	C22 hydroxylation		[90]
CYP724B1	85	724	*Oryza sativa*	AB158759	steroid	campesterol / campestanol	C22 hydroxylation	22*S*-hydroxy- campesterol / 6-deoxo- cathasterone	[62]
CYP724B2	85	724	*Solanum* *lycoper-* *sicum*	XM_004243170	steroid	campesterol / 24*R*-ergost-4- en-3-one / 24*R*-5α-ergos- tan-3-one / campestanol	C22 hydroxylation	22-hydroxy- campesterol / 22*S*-hydroxy- 24*R*-ergost- 4-en-3-one / 22*S*-hydroxy- 24*R*-5α- ergostan- 3-one / 6-deoxo- cathasterone	[63]
CYP94D108	86	94	*Paris polyphylla*	MK636703	steroid	16*S*-hydroxy- 22-oxo- cholesterol	C27 hydroxy- lation / spiro- ketalisation	diosgenin	[35]
CYP94D109	86	94	*Paris polyphylla*	MK636704	steroid	16*S*-hydroxy- 22-oxo- cholesterol	C27 hydroxy- lation / spiro- ketalisation	diosgenin	[35]
CYP94N1	86	94	*Veratrum californicum*	KJ869255	steroid	22*R*-hydroxy- cholesterol	C26 hydroxylation	22,26-di- hydroxy- cholesterol and 22-hydroxy- cholesterol- 26-al	[64]
CYP710A1	710	710	*Arabidopsis thaliana*	AB219423	steroid	β-sitosterol	C22 desaturation	stigmasterol	[91]
CYP710A2	710	710	*Arabidopsis thaliana*	AB233425	steroid	β-sitosterol / 24-epi- campesterol	C22 desaturation	stigmasterol/ brassicaster- ol	[91]
CYP710A4	710	710	*Arabidopsis thaliana*	NM_128444.2	steroid	β-sitosterol	C22 desaturation	stigmasterol	[91]
CYP710A11	710	710	*Solanum* *lycoper-* *sicum*	NM_001247585.2	steroid	β-sitosterol	C22 desaturation	stigmasterol	[91]

Probably the most well-known example of a triterpenoid-biased CYP family are the CYP716s (part of the CYP85 clan) [79], but also other families of the CYP85 clan such as CYP87, CYP85, or CYP90 contain mostly triterpene-modifying CYPs to date. The small clans CYP51 and CYP710 are other important examples of groups with a high preference for triterpenoid substrates. The highly diverse CYP71 clan, in contrast, only contains a few triterpene-modifying CYPs, particularly in the families CYP93, CYP712 and CYP705. The CYP72 family (CYP72 clan) also contains several known representatives. In other clans, however, not a single triterpene-modifying CYP has been identified so far, for example CYP97, CYP74, or CYP711.

The discovery of biosynthetic genes in plants often involves the screening of large pools of gene candidates derived from sequencing studies [93–96]. Hence, efficient approaches are needed to select the most promising gene candidates, particularly for large gene families such as CYPs. Our summarised phylogenetic distribution of known triterpenoid-modifying CYPs therefore might facilitate the discovery of new CYPs in triterpenoid and steroid pathways in plants by highlighting CYP families with a known propensity to participate in these pathways.

### Major reaction types of triterpenoid- and steroid-modifying CYPs

The basic polycyclic skeletons of triterpenoids and steroids are created by oxidosqualene cyclases (OSCs) from the universal substrate 2,3-oxidosqualene [5]. As different folding modes (chair–boat–chair vs chair–chair–chair) and different ring sizes can occur during this cyclisation cascade, resulting triterpene and sterol scaffolds have drastically different three-dimensional shapes. For this reason, CYPs are typically specific to a certain group of triterpenoid scaffolds. Hence, we summarised our list of 149 triterpenoid/steroid CYPs (Table 1) according to their target scaffold.

**Figure 3:**
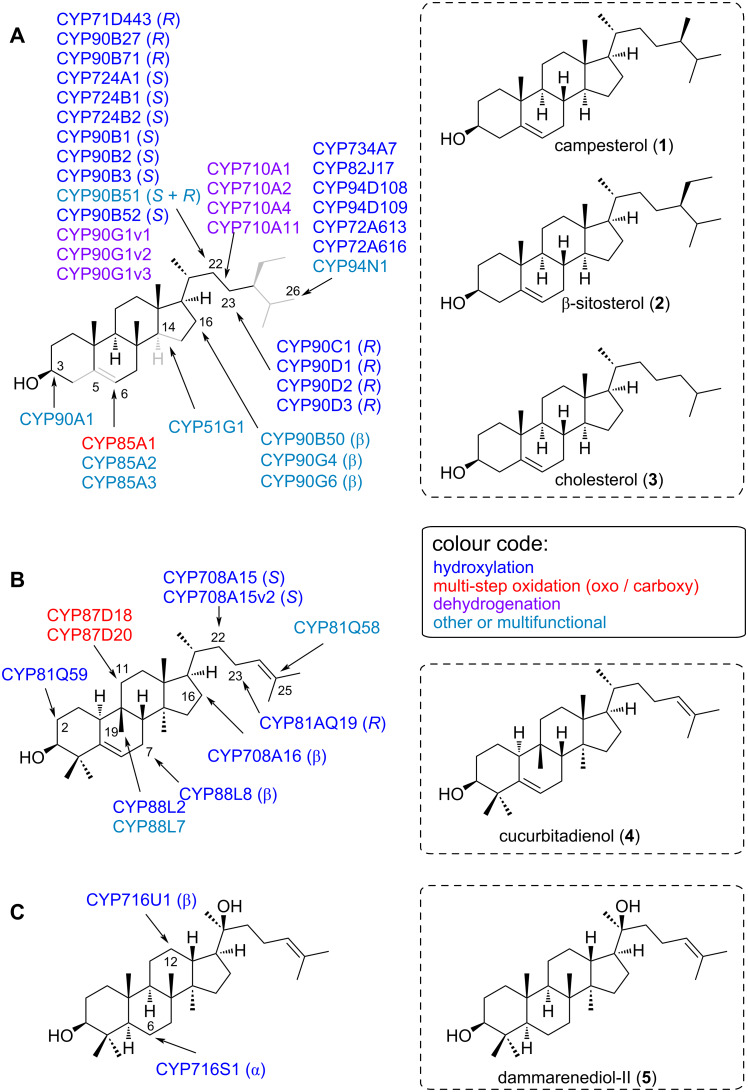
CYPs modifying steroid (A), cucurbitacin steroid (B) and tetracyclic triterpene (C) backbones. Substructures in grey indicate regions where major structural differences occur between different substrates of the same class. Representative substrate skeletons (not exact substrates) are shown in the dotted boxes. For exact substrate specificity see Table 1.

Figure 3 covers plant CYPs acting on steroid, cucurbitacin, or simple tetracyclic triterpenoid scaffolds. Important scaffolds here are campesterol (**1**), β-sitosterol (**2**), cholesterol (**3**), cucurbitadienol (**4**), and dammarenediol-II (**5**). Not surprisingly, CYPs involved in the biosynthesis of essential sterols in plants are highly conserved and play a crucial role in their growth and development. For example, members of the CYP710A subfamily were characterised as C22 desaturases in *Arabidopsis* and tomato [91,97]. Three CYPs, CYP710A1, CYP710A2 and CYP710A4 were identified in *Arabidopsis* and CYP710A11 was identified in tomato. All four CYPs could produce stigmasterol from β-sitosterol (**2**) in enzyme assays performed in vitro. However, *Arabidopsis* CYP710A2 showed substrate flexibility towards campesterol (**1**) epimers and could also produce brassicasterol from 24-epicampesterol in vitro. Enzymes of the CYP51G subfamily (CYP51 clan) function as sterol 14α-demethylases in green plants [24–25,98]. These enzymes catalyse oxidation of the C14α methyl group to trigger elimination of formic acid [24–25]. The sister subfamily CYP51H, on the other hand, is only found in monocots. AsCYP51H10 from *Avena sativa* (oat) is a multifunctional CYP that performs hydroxylation and epoxidation reactions of the β-amyrin (**6**) scaffold to produce 12,13β-epoxy-16β-hydroxy-β-amyrin [1,99]. Thus, CYP51H10 is an example of a neofunctionalised CYP recruited from primary sterol metabolism.

Two members of the CYP87D subfamily decorate the tetracyclic scaffold in plants from the Cucurbitaceae family (Figure 3B). CYP87D18 (CYP85 clan) was identified as a multifunctional C11 oxidase involved in the biosynthetic pathway of mogrosides. Mogrosides, isolated from ripe fruits of *Siraitia grosvenorii* (Cucurbitaceae) are glycosylated triterpenoid saponins with rare C24 and C25 hydroxylation [100]. Based on feeding assays in yeast it was found that CYP87D18 catalyses a two-step sequential C11 oxidation of cucurbitadienol (**4**) to 11-hydroxycucurbitadienol and 11-oxo-cucurbitadienol [101]. CYP87D18 also catalysed C11 hydroxylation of *trans*-24,25-dihydroxycucurbitadienol to form trihydroxylated mogrol in yeast [102].

**Figure 4:**
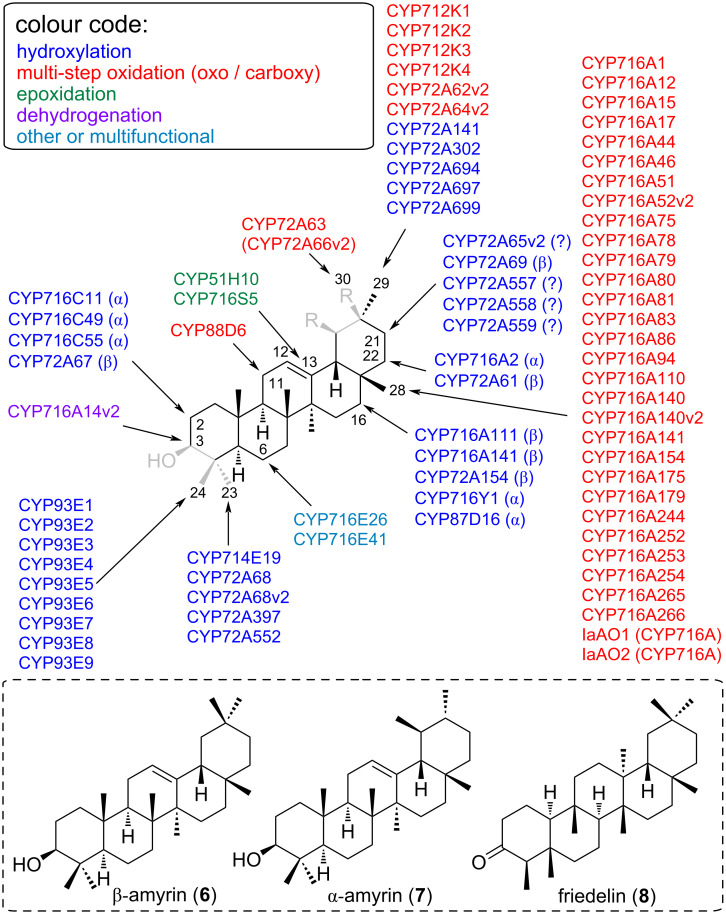
CYPs modifying pentacyclic 6-6-6-6-6 triterpenes. Substructures in grey indicate regions where major structural differences occur between different substrates of the same class. Representative substrate skeletons (not exact substrates) are shown in the dotted boxes. For exact substrate specificity see Table 1.

CYPs acting on pentacyclic 6-6-6-6-6 triterpenes, which include the extremely important and widespread scaffolds β-amyrin (**6**), α-amyrin (**7**), and friedelin (**8**), are summarised in Figure 4. Of particular relevance in this area is the CYP716 family, which plays a central role in the diversification of triterpenoids in eudicots [79]. Members of the CYP716A subfamily were mostly identified as C28 oxidases that catalyse three-step oxidation of α-amyrin (**7**), β-amyrin (**6**) and lupeol (**10**) to ursolic acid, oleanolic acid, and betulinic acid, respectively [71,73]. Nonetheless, other CYP716 enzymes have evolved to perform a wider range of modifications of triterpenoids; several CYP716 enzymes were found to catalyse C3 oxidation of α-amyrin (**7**) and β-amyrin (**6**), C16α oxidation of β-amyrin (**6**), or C22α oxidation of α-amyrin (**7**) [40,72,79]. Some members even act on triterpenoid scaffolds other than the 6-6-6-6-6 pentacyclic triterpenes. For example, two CYP716 enzymes from *Panax ginseng* act on tetracyclic scaffolds; CYP716U1 hydroxylates dammarenediol-II (**5**) to protopanaxadiol, and CYP716S1 hydroxylates the C6 of protopanaxadiol to form protopanaxatriol [76,103]. CYP716A113v1 from *Aquilegia coerulea* hydroxylates cycloartenol with unknown regiospecificity when expressed in a yeast strain harbouring a tomato cycloartenol synthase gene [79].

CYP712 family members (clan 71) were first identified in the biosynthetic pathway of nor-triterpenoid celastrol, a potent anti-obesity metabolite [42–43]. In two independent studies, transcriptome mining and functional studies in *Nicotiana benthamiana* were used to identify the CYPs CYP712K1, CYP712K2, CYP712K3, and CYP712K4 capable of oxidising friedelin (**8**) into polpunonic acid via an aldehyde intermediate [42–43].

Members of the CYP93E subfamily are restricted to legumes and are involved in the biosynthesis of triterpenoid saponins. So far, nine CYP93E members were identified from different legume species [37,40]. All of these perform C24 hydroxylation of β-amyrin (**6**) to form 24-hydroxy-β-amyrin. CYP93E1 also catalyses the conversion of sophoradiol to soyasapogenol B [37,40,46]. Members of other CYP93 subfamilies (CYP93A, B, C and G) are ubiquitous in flowering plants and are mostly involved in flavonoid biosynthesis [25–26].

**Figure 5:**
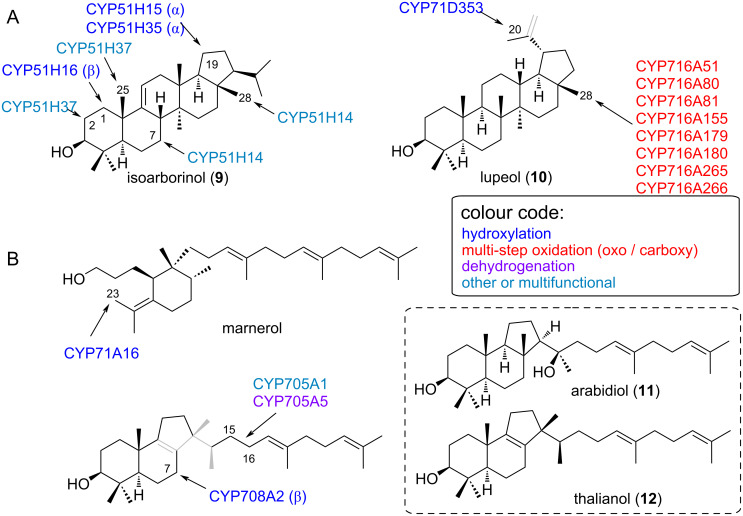
CYPs modifying pentacyclic 6-6-6-6-5 triterpenes (A) and unusual triterpenes (B). Substructures in grey indicate regions where major structural differences occur between different substrates of the same class. Representative substrate skeletons (not exact substrates) are shown in the dotted boxes. For exact substrate specificity see Table 1.

Lastly, CYPs acting on either pentacyclic 6-6-6-6-5 scaffolds, such as isoarborinol (**9**) or lupeol (**10**), or on unusual triterpene scaffolds such as arabidiol (**11**) or thalianol (**12**) are grouped in Figure 5. Enzymes from the CYP705 and CYP708 family catalyse Brassicaceae-specific reactions. The corresponding genes were found in operon-like gene clusters and catalyse the modification of monocyclic marnerol and tricyclic thalianol (**12**) in *Arabidopsis* [27,41]. Marneral synthase (MRN1) produces two oxidation products, one is marneral (aldehyde) and the other marnerol (alcohol). *Arabidopsis* CYP71A16 hydroxylates the allylic methyl side-chain of monocyclic marneral/marnerol to 23-hydroxymarneral/23-hydroxymarnerol. Modification of thalianol (**12**) involves CYPs from two clans. Genes encoding CYP708A2 (clan 85) and CYP705A5 (clan 71) are physically clustered with the thalianol synthase (THAS) gene, encoding the corresponding oxidosqualene cyclase. CYP708A2 oxidises the tricyclic thalianol (**12**) scaffold to 7β-hydroxythalianol, while CYP705A5 is a desaturase and introduces a double bond at C15 [41]. The related *Arabidopsis* CYP705A1 (also from clan 71) accepts a slightly different scaffold, arabidiol (**11**), triggering cleavage of the side chain at the same C15 instead of dehydrogenation. This shows that even closely related CYPs from the same subfamily can exhibit distinct differences in their substrate and reaction profiles.

### Recent examples of triterpenoid and steroid cytochrome P450 monooxygenases

**Figure 6:**
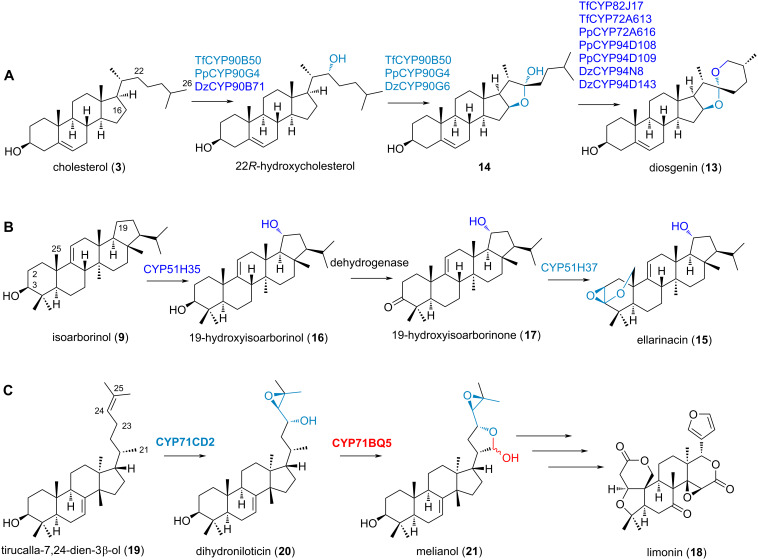
Recent examples of multifunctional CYPs in triterpenoid and steroid metabolism in plants that install complex oxidative modifications; in addition, their discovery also showcases modern approaches to elucidate plant specialised metabolism. A) CYPs from different plants producing diosgenin (**13**) (Tf: *Trigonella foenum-graecum*; Pp: *Paris polyphylla*; Dz: *Dioscorea zingiberensis*) [35,66]. B) Formation of the defence compound ellarinacin (**15**) in bread wheat [26]. Stereochemistry of ellarinacin (**15**) is shown as published. C) Biosynthesis of the key intermediate melianol (**21**) in the pathway to the limonoid limonin (**18**) [29]. The stereochemistry is shown as published.

In this last section, we will illustrate selected examples that showcase the enzymatic versatility of CYPs in plant triterpenoid and steroid metabolism (Figure 6).

Diosgenin (**13**) is a specialised plant natural product with a unique 5,6-spiroketal moiety that serves as an inexpensive raw material for the industrial synthesis of steroidal drugs. Diosgenin (**13**) biosynthesis from cholesterol (**3**) was explored in *Paris polyphylla* (Pp; monocot), *Trigonella foenum-graecum* (Tf; dicot) and *Dioscorea zingiberensis* (Dz; monocot) (Figure 6A) [35,66]. Multifunctional CYPs PpCYP90G4/TfCYP90B50 were independently recruited from the ancient CYP90B subfamily involved in brassinosteroid biosynthesis to catalyse the initial C22,16 dihydroxylation of cholesterol (**3**) [35]; in contrast, the related CYP DzCYP90B71 was found to catalyse only the first hydroxylation at C22 [66]. This step is followed by a rate-limiting cyclisation step through unstable furostanol intermediate **14** that involves CYP-catalysed oxidative ring closure, leading to a hemiketal bridge between C16 and C22. Following these initial hydroxylations, CYPs from multiple families catalyse end-of-chain hydroxylation at C27 which is followed by spontaneous spiroketalisation to form diosgenin (**13**). The CYP pairs PpCYP90G4*-*PpCYP94D108 in *P. polyphylla* and TfCYP90B50*-*TfCYP82J17 in *T. foenum-graecum* resulted in the highest diosgenin (**13**) production. Diosgenin (**13**) biosynthesis in distantly related plants is an example of catalytic plasticity embedded within the ancient CYP90Bs. Especially CYPs from large families often show high substrate promiscuity which facilitates duplication events resulting in neofunctionalisation [15].

Ellarinacin (**15**) is a defence-related arborinane-type triterpenoid that was recently discovered in bread wheat (*Triticum aestivum*) by genome mining (Figure 6B) [26]. The ellarinacin gene cluster encodes the three CYP enzymes TaCYP51H35, TaCYP51H37 and TaCYP51H13P, with the latter carrying a premature stop codon. TaCYP51H35 catalyses the C19-hydroxylation of isoarborinol (**9**) to form 19-hydroxyisoarborinol (**16**), which is oxidised to ketone **17** by a dehydrogenase (TaHID). TaCYP51H37 then carries out a remarkable double oxidation at the methyl group C25 as well as C2, leading to the highly unusual acetal-epoxide proposed for ellarinacin (**15**). This work therefore not only represents an important example how a CYP51H evolved by gene duplication and neofunctionalisation from a sterol biosynthetic gene, but also demonstrates the capacity of CYPs to catalyse unique enzymatic cascades.

Limonoids are highly oxidised, modified and truncated triterpenoids; one of the most well-known limonoids is the eponymous compound limonin (**18**), which contributes to the bitter taste of citrus products (Figure 6C) [104]. The first steps of limonoid biosynthesis were recently explored by functional characterisation in heterologous hosts [29,105–107]. There, CYP enzymes MaCYP71CD2 and MaCYP71BQ5 from *Melia azedarach* initiate the ring formation on the side chain of the triterpene precursor tirucalla-7,24-dien-3β-ol (**19**) in a sequential manner. MaCYP71CD2 is a bifunctional CYP that hydroxylates C23 and additionally introduces a C24–C25 epoxide on the side chain of tirucalla-7,24-dien-3β-ol (**19**), yielding dihydroniloticin (**20**). MaCYP71BQ5 then oxidises the methyl group C21 to a formyl group, leading to spontaneous hemiacetal ring formation in the product melianol (**21**). It is believed that these transformations are the starting point for formation of the characteristic furan ring of limonoids [29].

Taken together, these case studies not only represent impressive examples how CYPs create chemical complexity in plant triterpenoid and steroid metabolism, but also illustrate state-of-the-art approaches to discover and characterise new CYPs by genome mining, co-expression analyses, and efficient heterologous expression systems.

## Conclusion

In this review, we provided a comprehensive overview over the phylogenetic distribution and diverse metabolic reactions catalysed by CYPs involved in the tailoring of triterpenoids and steroids from plants, covering 149 CYPs that have been functionally characterised to date (Table 1). Considering that up to 1% of all plant genes encode CYPs and that triterpenoids are one of the largest natural product classes in plants, we expect that this number will rise quickly in years to come. Several of our examples highlight the substrate promiscuity embedded within ancient CYP families, which enables rapid functional extension to acquire unique catalytic functions during duplication events [15,26,79]. The increasing availability of high-quality transcriptome and genome data even of non-model plants together with reliable and efficient expression systems in yeast and in *Nicotiana benthamiana* will facilitate future approaches to fully harness the diversity of triterpenoids and steroids found in plants. In combination with ground-breaking machine learning approaches for protein structure prediction such as AlphaFold2 [108], we anticipate that the catalytic repertoire of CYPs will be exploited much more for the biotechnological production of tailor-made triterpenoids and steroids in the near future. We hope that our review provides a good starting point for such further studies.

## Supporting Information

File 1 High-quality version of the phylogenetic tree shown in Figure 2 with tip labels.
