# Supplementary material for: Cytochrome P450 monooxygenase-mediated tailoring of triterpenoids and steroids in plants
Source: Beilstein J Org Chem. 2022 Sep 21;18:1289–310. doi: 10.3762/bjoc.18.135 (PMC9520826; doi:10.3762/bjoc.18.135)
Supplement: File 1 — High-quality version of the phylogenetic tree shown in Figure 2 with tip labels. [file Beilstein_J_Org_Chem-18-1289-s001.pdf]

BEILSTEIN JOURNAL OF ORGANIC CHEMISTRY

Supporting Information

for

Cytochrome P450 monooxygenase-mediated tailoring of triterpenoids and steroids in plants

Karan Malhotra and Jakob Franke

Beilstein J. Org. Chem. **2022,** *18,* 1289–1310. doi:10.3762/bjoc.18.135

High-quality version of the phylogenetic tree shown in Figure 2 with tip labels

License and Terms: This is a supporting information file under the terms of the Creative Commons Attribution License (https://creativecommons.org/licenses/by/4.0). Please note that the reuse, redistribution and reproduction in particular requires that the author(s) and source are credited and that individual graphics may be subject to special legal provisions.
The license is subject to the *Beilstein Journal of Organic Chemistry* terms and conditions: (https://www.beilstein-journals.org/bjoc/terms)

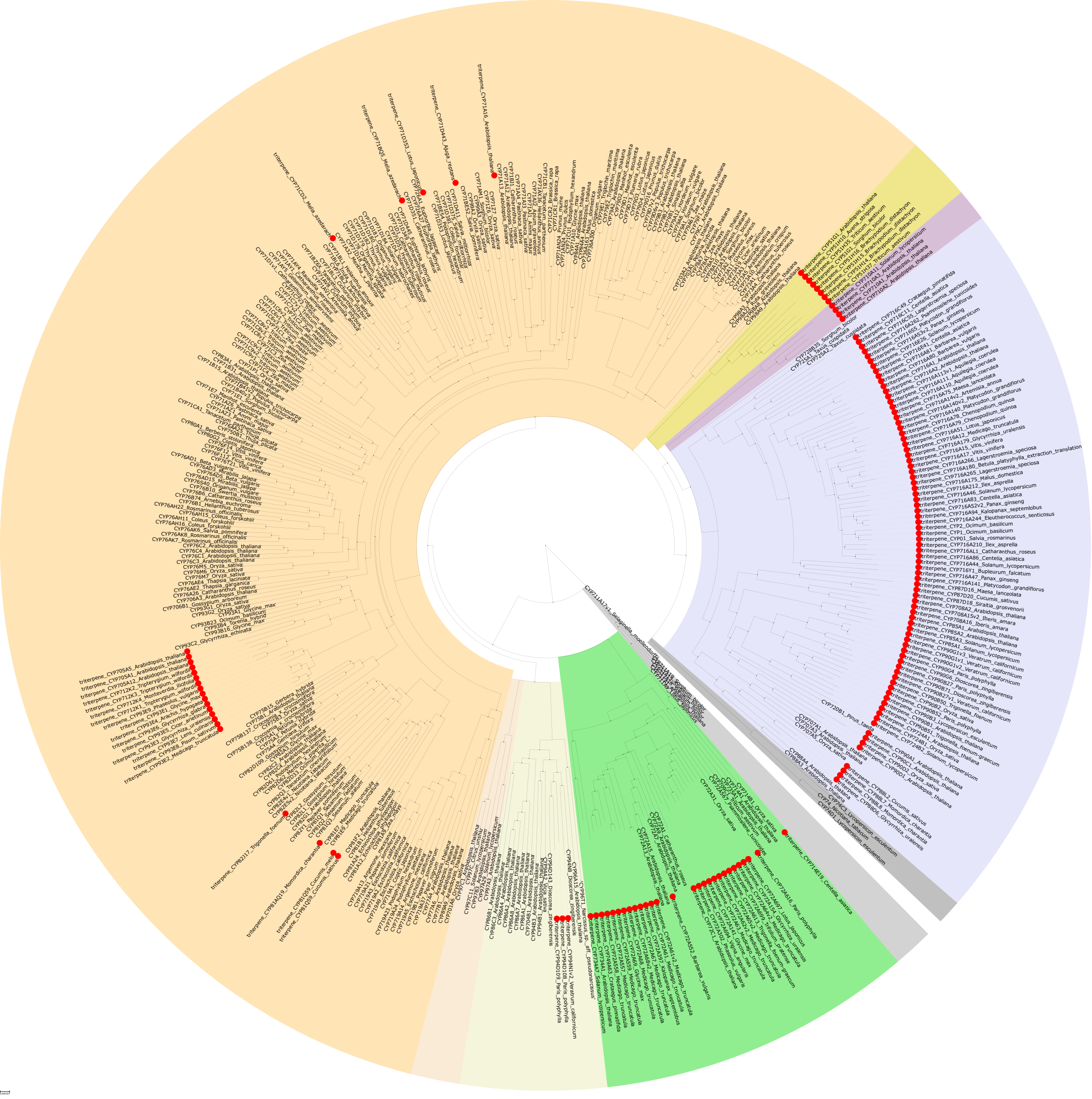

triterpene_CYP716A17_Vitis_vinifera
triterpene_CYP716A266_Lagerstroemia_speciosa
triterpene_CYP716A180_Betula_platyphylla_extraction_translation
triterpene_CYP716A265_Lagerstroemia_speciosa
triterpene_CYP716A175_Malus_domestica
triterpene_CYP716A212_Ilex_asprella
triterpene_CYP716A46_Solanum_lycopersicum
triterpene_CYP716A83_Centella_asiatica
triterpene_CYP716A52v2_Panax_ginseng
triterpene_CYP716A94_Kalopanax_septemlobus
triterpene_CYP716A244_Eleutherococcus_senticosus
triterpene_CYP2_Ocimum_basilicum
triterpene_CYP1_Ocimum_basilicum
triterpene_CYP01_Salvia_rosmarinus
triterpene_CYP716A210_Ilex_asprella
triterpene_CYP716AL1_Catharanthus_roseus
triterpene_CYP716A86_Centella_asiatica
triterpene_CYP716A44_Solanum_lycopersicum
triterpene_CYP716Y1_Bupleurum_falcatum
triterpene_CYP716A47_Panax_ginseng
triterpene_CYP716A141_Platycodon_grandiflorus
triterpene_CYP87D20_Cucumis_sativus
triterpene_CYP87D16_Maesa_lanceolata
triterpene_CYP87D18_Siraitia_grosvenorii
triterpene_CYP708A2_Arabidopsis_thaliana
triterpene_CYP708A15v2_Iberis_amara
triterpene_CYP708A16_Iberis_amara
triterpene_CYP85A1_Arabidopsis_thaliana
triterpene_CYP85A2_Arabidopsis_thaliana
triterpene_CYP85A3_Solanum_lycopersicum
triterpene_CYP85A1_Solanum_lycopersicum
CYP76C2_Arabidopsis_thaliana
CYP76C4_Arabidopsis_thaliana
CYP76C1_Arabidopsis_thaliana
CYP76C3_Arabidopsis_thaliana
CYP76M5_Oryza_sativa
CYP76M6_Oryza_sativa
CYP76M7_Oryza_sativa
CYP76AE4_Thapsia_laciniata
CYP76AE2_Thapsia_garganica
CYP76A26_Catharanthus_roseus
CYP706A3_Arabidopsis_thaliana
CYP706B1_Gossypium_arboreum
CYP93G1_Oryza_sativa
CYP93G2_Oryza_sativa
CYP93A1_Glycine_max
CYP93B23_Ocimum_basilicum
CYP93B4_Torenia_hybrid
CYP93B16_Glycine_max
CYP93C2_Glycyrrhiza_echinata
